# Serum Predose Metabolic Profiling for Prediction of Rosuvastatin Pharmacokinetic Parameters in Healthy Volunteers

**DOI:** 10.3389/fphar.2021.752960

**Published:** 2021-11-12

**Authors:** Anne Michelli Reis Silveira, Gustavo Henrique Bueno Duarte, Anna Maria Alves de Piloto Fernandes, Pedro Henrique Dias Garcia, Nelson Rogerio Vieira, Marcia Aparecida Antonio, Patricia de Oliveira Carvalho

**Affiliations:** ^1^ Health Sciences Postgraduate Program, São Francisco University–USF, Bragança Paulista, Brazil; ^2^ Integrated Unit of Pharmacology and Gastroenterology (UNIFAG), São Francisco University–USF, Bragança Paulista, Brazil

**Keywords:** personalized medicine, metabolomics, rosuvastatin, prediction, machine learning, pharmacometabolomics

## Abstract

Rosuvastatin is a well-known lipid-lowering agent generally used for hypercholesterolemia treatment and coronary artery disease prevention. There is a substantial inter-individual variability in the absorption of statins usually caused by genetic polymorphisms leading to a variation in the corresponding pharmacokinetic parameters, which may affect drug therapy safety and efficacy. Therefore, the investigation of metabolic markers associated with rosuvastatin inter-individual variability is exceedingly relevant for drug therapy optimization and minimizing side effects. This work describes the application of pharmacometabolomic strategies using liquid chromatography coupled to mass spectrometry to investigate endogenous plasma metabolites capable of predicting pharmacokinetic parameters in predose samples. First, a targeted method for the determination of plasma concentration levels of rosuvastatin was validated and applied to obtain the pharmacokinetic parameters from 40 enrolled individuals; then, predose samples were analyzed using a metabolomic approach to search for associations between endogenous metabolites and the corresponding pharmacokinetic parameters. Data processing using machine learning revealed some candidates including sterols and bile acids, carboxylated metabolites, and lipids, suggesting the approach herein described as promising for personalized drug therapy.

## Introduction

Rosuvastatin is a lipid-lowering agent extensively used to treat hypercholesterolemia and prevent progression of coronary artery diseases. It is well known for increasing serum levels of high-density lipoprotein (HDL) cholesterol (McTaggart and Jones, 2008) and decreasing low-density lipoprotein (LDL) cholesterol (Betteridge and Gibson, 2007) and triglycerides (Crouse, 2008) by competitively inhibiting the enzyme 3-hydroxy-3-methylglutaryl–coenzyme A (HMG-CoA) reductase, the rate-limiting enzyme involved in cholesterol biosynthesis (Olsson et al., 2002). It is biotransformed to two metabolites: rosuvastatin-5S lactone (inactive metabolite) and N-desmethyl rosuvastatin (active metabolite) primarily by CYP2C9 (cytochrome P450) and, to a lesser extent, by CYP 2C19 and CYP 3A4 isoenzymes. Previous studies demonstrate that rosuvastatin-5S-lactone and N-desmethyl rosuvastatin concentrations were found to be much lower than those of rosuvastatin. The mean maximum plasma drug concentration (*C*_max_) values for rosuvastatin-5S-lactone were 12%–24% lower than those for rosuvastatin and <10% for N-desmethyl rosuvastatin. Moreover, when considering the sum of the parent compound and active metabolites, drug-interaction studies suggest that rosuvastatin accounts for 87% of circulating active HMG-CoA reductase inhibitors (Martin et al., 2003). Although statins are usually well tolerated, adverse effects associated with statin treatment include skeletal muscle toxicity, myopathy (Thompson et al., 2003), or even rhabdomyolysis in rare cases (Wooltorton, 2004), and they decrease adherence to therapeutic regimens. There is evidence of a substantial inter-individual variability in statin responses as well as in their pharmacokinetics in humans (Lee et al., 2005; Pasanen et al., 2007; Keskitalo et al., 2009). Such variability results in drugs not reaching or not binding to their designated targets. Moreover, an increased statin plasma concentration can affect drug therapy safety by causing the adverse drug effects mentioned above.

Usually, it is reasonable to associate the variations among individual responses in drug therapy (from increased sensitivity to tolerance to the drug) with several key factors including epigenetic factors like genetic polymorphisms (Weinshilboum, 2003); demographic factors like age, sex, bodyweight, race, and ethnicity; environmental factors like diet, addictions (smoking, alcohol, etc.), previous/current single/multiple drug administration; and individual factors like accompanying diseases, disease status, microbiome, etc. (Li and Jia, 2013; Alomar, 2014). For rosuvastatin, a vast amount of literature points to a series of factors that may exert some influence on the therapeutic response of this statin. These factors may be divided into genetic and environmental factors. The most described genetic factor influencing rosuvastatin pharmacokinetics is associated with genes encoding the hepatic organic anion transporting polypeptide 1B1 (OATP1B1) and the ATP-binding cassette transporter G2 (*ABCG2*) proteins. Genetic polymorphisms influence *C*_max_ and area under curve (AUC) parameters (Pasanen et al., 2007; Keskitalo et al., 2009; Wan et al., 2015) in a manner sometimes affected also by factors such as ethnicity (Choi et al., 2008; Lee et al., 2013; Birmingham et al., 2015a; Birmingham et al., 2015b; Liu et al., 2016) and age (Kim et al., 2019). Multiple-drug intake also influences rosuvastatin response and pharmacokinetics (Schneck et al., 2004; Simonson et al., 2004; van der Lee et al., 2007; Busti et al., 2008; He et al., 2008; Samineni et al., 2012; Martin et al., 2016; Son et al., 2016, among others). The same influence is also observed with natural products, such as baicalin (Fan et al., 2008); epigallocatechin-3-gallate, the major component of green tea (Kim et al., 2017); and theaflavins, derived from black tea (Kondo et al., 2019). High-fat and high-calorie meals resulted in a reduction of rosuvastatin exposure and a decrease in absorption rate of rosuvastatin (Chen et al., 2020), showing the relevance of diet. Sex (Nazir et al., 2015) and drug formulation (Balakumar et al., 2013; Dudhipala and Veerabrahma, 2017) are other listed factors that influence pharmacokinetics and/or response to rosuvastatin.

Despite the fact that, in the field of medicine, drugs are usually described on the basis of the uniformity of the inter-individual responses, clinicians have become increasingly aware that the variability in drug therapy effectiveness is a direct result of the variability of drug response among different individuals. Thus, there is an urgent need to select the right prescription according to the patient’s characteristics to avoid drug therapy failure and adverse effects (Balashova et al., 2018). Personalized medicine proposes a tailored drug treatment to achieve maximal therapeutic effects with minimal adverse effects (Schork, 2015). The main idea is that clinical, laboratory, and individual characteristics like age, body weight, comorbidity, family history, biochemical laboratory parameters, etc. may be useful for personalizing drug therapy (Kitsios and Kent, 2012). Nowadays, those characteristics are already being used as predictors of inter-individual variability in drug responses and are called covariates (Joerger, 2012). A previous research has investigated the ability of covariates to predict inter-individual variability in rosuvastatin concentration. Using multiple linear regression modeling, results have revealed gender, age, body mass index, ethnicity, dose, and time from last dose as the best clinical predictors (DeGorter et al., 2013).

Since genetic polymorphisms are partly responsible for individual variations in drug therapy, pharmacogenomics can also be useful in predicting them. However, drug therapy effectiveness relies on the efficiency of the drug’s reaching and binding to its designated target, and those required steps are also affected by the individual phenotype, so pharmacogenomics and conventional covariates have a limited reach. Due to the intrinsic difficulty to predict inter-individual variation, a research on phenotypic markers associated with rosuvastatin inter-individual variation would be tremendously valuable for determining optimal drug therapy and minimizing adverse effects.

Given that metabolomics is the closest omics science to the phenotype, it is extremely useful to obtain a picture of the ongoing physiological condition of a given individual in order to provide clinicians useful information about drug selection and dosing predictions (Kantae et al., 2017). Metabolites are the final products of metabolism and are the result of complex downstream relations among genes, proteins, and environment, being, therefore, among the current omics sciences, the most integrative approach regarding real-time physiological condition assessment. Its newest, emergent branch, the so-called pharmacometabolomics, employs metabolomic strategies for pharmacotherapy personalization by measuring endogenous metabolites related to drug variability, thereby obtaining insights about the interplay between individual physiology and drug pharmacology (Kantae et al., 2017; Balashova et al., 2018). Since it was first applied in rat urine predose samples to establish an association between endogenous metabolites and pharmacokinetic/pharmacodynamic parameters of paracetamol (Clayton et al., 2006), many research efforts have successfully employed pharmacometabolomics for predictive purposes. The main applications using a pharmacometabolomic strategy in practical medicine have been with drugs like paracetamol (Clayton et al., 2009; Winnike et al., 2010), tacrolimus (Phapale et al., 2010), simvastatin (Kaddurah-Daouk et al., 2011a; Trupp et al., 2012), and atorvastatin (Huang et al., 2015). Moreover, studies involving drug-metabolizing enzyme activity have investigated enzymes like CYP3A4/5 (Diczfalusy et al., 2011), CYP3A4 (Rahmioglu et al., 2011), CYP3A (Shin et al., 2013), and CYP2D6 (Tay-Sontheimer et al., 2014) and diseases, including colorectal cancer (Backshall et al., 2011), depression (Kaddurah-Daouk et al., 2011b), lymphoid malignances (Muhrez et al., 2017), and so on.

This work focuses on applying pharmacometabolomic strategies using ultra performance liquid chromatography–quadrupole time-of-flight mass spectrometry (UPLC-QTOF-MS^E^) in healthy human plasma samples to predict the main pharmacokinetic parameters, AUC and the *C*_max_, based on endogenous molecules in the predose baseline. First, we validated a liquid chromatography coupled to low-resolution mass spectrometry (LC-MS/MS) method for rosuvastatin quantification in plasma samples from volunteers to whom a rosuvastatin reference formulation was administered to obtain their corresponding drug concentrations and calculate the pharmacokinetic parameters. Then, predosing samples were analyzed by UPLC-QTOF-MS^E^ (where E represents collision energy) applying an untargeted metabolomic profiling strategy. Multivariate statistical modeling was employed to search for associations between metabolite profiling and the investigated pharmacokinetic parameters to find relevant metabolites responsible for predicting individual AUC and *C*_max_ values of the enrolled healthy volunteers. Our results have proved the value and effectiveness of the employed strategy in predicting individual pharmacokinetic outcomes. So far, to the best of our knowledge, there has been no previous report of the application of untargeted metabolomics in predose human plasma samples to predict AUC and *C*_max_ of rosuvastatin, making this a pioneering effort in the field.

## Experimental Method

### Chemicals and Reagents

Rosuvastatin calcium was purchased from European Pharmacopoeia. Internal standard (IS) diazepam was purchased from Fundação Oswaldo Cruz. Bromo-l-phenylalanine was purchased from Sigma-Aldrich, and all other reagents were purchased from Merck KgaA (Darmstadt, Germany). Diazepam was chosen as IS due to the satisfactory results regarding molecular stability, chromatographic peak shape, molecular extraction recovery, analytical accuracy and precision obtained during the preliminary tests, method validation, and application.

### Healthy Human Volunteers and Pharmacokinetic Study Design

Forty adult volunteers of both sexes, aged between 18 and 50 with a body mass index between 18.5 and 29.9 kg/m^2^, were selected for the study after assessment of their health status by clinical evaluation (physical examination and electrocardiogram) and laboratory tests. The set of laboratory tests is listed in the Supplementary Material. The protocol complied with the current Brazilian legislation on clinical research in humans and was approved by the Research Ethics Committee, duly authorized by the National Health Council, and under the guidelines of Good Clinical Practice (Brazil Platform CAAE: 26762719.9.0000.5514, registered on July 15, 2020—https://plataformabrasil.saude.gov.br/login.jsf). All subjects gave their written informed consent and were free to withdraw from the study at any time.

The reference formulation was 20-mg rosuvastatin calcium tablets (Crestor^®^ 20 mg—AstraZeneca do Brasil Ltda.), with administration of one single-dose tablet. During each period, the volunteers were hospitalized at 7:30 p.m. and had a supper before 9:00 p.m. After an overnight fast, they received (at ∼8:00 a.m.) a tablet of rosuvastatin calcium (20 mg). Water (200 ml) was given immediately after the drug administration, and the volunteers then fasted for 4 h, after which period a standard lunch was served. No other food was allowed during the “in house” period, but liquid consumption was permitted *ad libitum* 2 h after tablet administration (with the exception of xanthine-containing drinks, including tea, coffee, and cola). At 2 h before and 5 and 36 h after the dose administration, systolic and diastolic arterial pressure (measured non-invasively with a sphygmomanometer), heart rate, and temperature were recorded. The hospitalization period was 84 h.

Blood samples (8 ml) from a suitable antecubital vein were collected by indwelling catheter into EDTA-containing tubes at 0, 1.00, 1.50, 2.00, 2.50, 3.00, 3.33, 3.67, 4.00, 4.33, 4.67, 5.00, 5.50, 6.00, 8.0, 10.0, 12.0, 24.0, 48.0, and 72.0 h postdosing. The blood samples were centrifuged at ∼2,000×*g* for 10 min at 4°C, and the plasma was stored at −70°C until assayed.

### LC-MS/MS Method Validation for Analysis of Rosuvastatin Concentrations

#### Liquid Chromatography

Chromatographic separations were performed in an LC-20AD analytical pump (Shimadzu) using a SIL-20A HT autosampler (Shimadzu). A Luna 5-μm C18 100A 150 × 4.6 mm column (Phenomenex) was employed as stationary phase, and acetonitrile (B) and water with 0.1% formic acid (A) were employed as mobile phase. The mobile phase flow rate comprised of 65% of B was set at 1.7 ml min^−1^, resulting in a total run of 2.8 min. Autosampler temperature was set at 22°C, while the injection volume was 20 µl.

#### Mass Spectrometry

Mass spectrometry analyses were performed using a Quattro Micro (Micromass) mass spectrometer equipped with an electrospray ionization (ESI) source. Analyses were performed in positive ionization mode using nitrogen as desolvation gas. ESI source parameters were set as follows: source temperature of 105°C, desolvation temperature of 480°C, desolvation flow of 800 l h^−1^, and capillary voltage of 3 kV. Rosuvastatin was detected by a multiple reaction monitoring (MRM) transition of 482.18 > 258.14 using a cone voltage of 35 V, and diazepam was detected by a MRM transition of 285.20 > 193.30 using a cone voltage of 30 V. Data were acquired using MassLynx 4.1.

#### Preparation of Standards and Quality Controls

A stock solution of rosuvastatin was prepared by accurately weighing and dissolving the standard in acetonitrile/water (80:20 *v*/*v*) to a concentration of 100 μg ml^−1^. Then, the solution was sequentially diluted to working solutions of 1, 0.152, and 0.0024 μg ml^−1^. IS diazepam was dissolved in acetonitrile/water (80:20 *v*/*v*) to obtain a 100-μg ml^−1^ stock solution and then diluted to obtain a 0.015-μg ml^−1^ working solution. Method validation, calibration curve, and quality control analysis were carried out using blank plasma calibration standards including 0.2, 1, 5, 10, 20, 30, 40, and 50 ng ml^−1^ blanks and quality control (QC) of 0.6, 25.0, and 38 ng ml^−1^ samples prepared by spiking the above-described working solutions of rosuvastatin and IS in a blank biological matrix. Stock and working solutions were stored in a refrigerator and allowed to equilibrate (30 min) at room temperature before use.

#### Sample Extraction

Three hundred microliters of plasma were aliquoted into a microcentrifuge tube. Then, 25 µl of IS solution were added to the plasma. Next, 25 µl of HCl 1 M as well as 1,000 µl of a diethyl ether/dichloromethane (70:30 *v*/*v*) were added to the same tube. The content was shaken for 5 min and then centrifuged for 10 min, at 132,000, at 4°C. After that, the supernatant was transferred to another tube and dried under nitrogen stream. An acetonitrile/water solution (80:20 *v*/*v*) was used for resuspension of the dried content, which was shaken and finally transferred to an insert before injection.

#### Method Validation

The analytical method was validated for the following parameters: specificity, carryover, matrix effect, calibration curve, accuracy, precision, and stability.

Specificity was ascertained by analyzing blank human plasma samples from six individuals and comparing the chromatograms with chromatograms from blank human plasma spiked with rosuvastatin in the lower limit of quantification (LLOQ) concentration and IS.

Carryover was ascertained by analyzing three injections of the same blank sample, one before and two after an injection of a sample in the upper limit of quantification (ULOQ) concentration. Results were compared with LLOQ chromatograms.

Matrix effect was ascertained by spiking eight different extracted blank human plasma samples with rosuvastatin at QC concentrations and the IS. Peak areas of extracted spiked samples were compared to those of standard solutions.

The calibration curve was prepared using six different blank human plasma samples (three for weighting tests and three for linearity tests) spiked with concentrations at eight levels ranging from 0.2 to 50 ng ml^−1^. Analyte concentrations in samples were calculated by linear regression equation, typically described by equation *y* = *ax* + *b* where *y* corresponds to the analyte/IS peak-area ratio and *x* corresponds to the ratio of rosuvastatin to IS concentration. Due to the range of the calibration curve and lower value of the sum of the relative errors of the nominal values of the calibration versus its values obtained by the curve equation, the weighting factor of reciprocal concentration squared (1/*x* ^2^) was applied.

Intra- and inter-batch accuracy and precision were evaluated at three different levels (0.6, 25, and 38 ng ml^−1^) of QC samples in quintuplicate in three different batches. The accuracy of the method was expressed as relative error (RE), whereas precision was obtained by calculating the within- and between-run coefficient of variation (CV). The acceptance criteria for each quality control were that CV must not exceed 15% for QC and 20% for LLOQ.

Freezing and thawing stabilities for rosuvastatin in human plasma samples were ascertained after four cycles and the analytical process conducted at low and high QC concentrations. Samples were frozen at −70°C in four cycles of 12, 24, 36, and 48 h. Autosampler stability was investigated over a 24-h storage period in the autosampler tray with low and high quality control concentrations. Long-term stability was also evaluated over a 160-day storage period at −70°C. The acceptance criteria for each quality control were that CV and accuracy must not exceed 15%.

#### Determination of Rosuvastatin Concentrations in Human Plasma Samples and Pharmacokinetic and Statistical Analysis

The validated LC-MS/MS method was applied to measure rosuvastatin concentrations to obtain additional pharmacokinetic parameters.

Pharmacokinetic parameters were calculated from plasma levels employing a non-compartmental statistic using WinNon-Lin 8.3 software (Pharsight, United States). Following Food and Drug Administration (FDA) guidelines*,* blood samples were drawn up to a period of three to five times the terminal elimination half-life (*t*_1/2_) and the mean AUC_0–t_/AUC_0–∞_ ratio was required to be higher than 80%. The area under the concentration–time curve (AUC_0–t_) was calculated by the trapezoidal method. The total area under the curve (AUC_0–∞_) was obtained up to the last measurable concentration, and extrapolations were performed using the last measurable concentration and the terminal elimination rate constant (*K*_e_). The terminal elimination rate constant, *K*_e_, was estimated from the slope of the terminal log_10_ transformed exponentially and multiplied by −2.303 phase of the plasma of rosuvastatin concentration–time curve (by means of the linear regression method) adjusted in the three last values. The terminal elimination half-life, *t*_1/2_, was then obtained as 0.693/*K*_e_. The *C*_max_ and the time to reach maximum plasma concentration (*T*_max_) values were determined by visual inspection of the plasma rosuvastatin concentration–time profiles. Results are presented as mean ± standard deviation (SD).

### UPLC-QTOF-MS^E^-Based Untargeted Metabolic Profiling for AUC and *C*_max_ Predictions

#### Liquid Chromatography

Chromatographic analyses were performed in an ACQUITY FTN H Class (Waters) liquid chromatograph. Separations were carried out using an ACQUITY CSH C18 column (Waters) with dimensions 2.1 × 100 mm × 1.7 µm as stationary phase using acetonitrile (B) and water with 0.1% formic acid (A) as mobile phase. Flow rate was set at 0.4 ml min^−1^. Segmented gradient was applied as follows: 0–2 min, 10% of B; 7 min, 90% of B kept for 2 min; and 9–11 min returning to initial conditions and kept for 2 min for column re-equilibration resulting in a 13-min run. Autosampler temperature was set at 20°C, whereas the injection volume was 0.5 µl for positive mode and 5 µl for negative mode.

#### Mass Spectrometry

High-resolution mass spectrometry analyses were performed using a XEVO-G2XSQTOF (Waters) equipped with an ESI source. Analyses were performed in both positive and negative ionization mode using nitrogen as the desolvation gas. ESI source parameters were set as follows: source temperature of 140°C, desolvation temperature of 550°C, desolvation flow of 900 l h^−1^, capillary voltage of 3 kV, sampling cone of 30 kV, and cone gas flow of 10 l h^−1^ for positive mode and capillary voltage of 2.5 kV, sampling cone of 40 kV, cone gas flow of 50 l h^−1^ for negative mode.

Spectra were acquired within an acquisition mass range of 50–1,700 Da using a data-independent MS^E^ approach. MS^E^ fragmentation settings were applied using 6 V of collision energy for low-energy scan and a 15–30-V ramp for high-energy scan. Leucine encephalin (Tyr-Gly-Gly-Phe-Leu, formula C_28_H_37_N_5_O_7_, molecular weight = 555.2693 g mol^−1^ at 200 pg μl^−1^ in acetonitrile/water 1:1 *v*/*v*) was used as lockmass to ensure exact mass measurements during data acquisitions. A 0.5-mM sodium formate solution was used for instrument calibration. MassLynx 4.1 was used for mass spectrometer control and data acquisition management. Samples were randomly analyzed. Pooled quality control samples were analyzed at the beginning of the batch in order to equilibrate the system as well as sample injections at regular intervals during the batch to monitor instrumental drifts that may occur. Additionally, at the end of the batch, serially diluted QC samples were analyzed in order to verify features that follow dilution trends and filtered.

#### Sample Extraction

A 50-µl aliquot of each sample was taken, and 100 µl of methanol containing the IS *p*-bromo-phenylalanine (200 µM) were added, both in the same centrifuge tube. Then, samples were shaken for 10 min for protein precipitation and centrifuged at 14,000 rpm for 1 min at 8°C. Finally, 100 µl of supernatant was transferred to a vial for instrumental analysis. Samples were randomly prepared. A pooled QC sample was prepared by mixing equal small aliquots of each study sample, distributed equally in regular intervals across the sample preparation batch, and extracted as such. In addition, five-level serially diluted QC samples were prepared.

#### Data Processing, Statistical Analysis, and Metabolite Identification

Raw data acquired by UPLC-QTOF-MS^E^ were directly imported to Progenesis QI (Nonlinear Dynamics) for peak detection and deconvolution, alignment, retention time correction, data filtering, and MS^E^-based ion annotation and identification to generate a suitable data matrix table for statistical analysis. For the feature-identification step, MassBank of North America (MoNA)-containing metabolomics libraries such as HMDB, LipidMaps, LipidBlast, and MassBank were used.

QC sample acquisition allowed for quality data checking and cleaning, and molecular features that showed relative standard deviation (RSD) > 25% after data cleaning and normalization were stripped out from the multivariate data modeling.

Machine learning modeling was performed by means of Elastic Net (sklearn.linear_model.ElasticNet), with the linear regression with combined L1 and L2 penalty priors as a regularizer, which penalizes models based on their complexity favoring simpler and more generalizing models. This algorithm was applied as estimator for AUC and *C*_max_ predictions using Python 3.7.10 with *scikit-learn* (Pedregosa et al., 2012) version 0.22.2. Other Python programing standard packages for data manipulation, such as *Pandas* (McKinney, 2010) and *Numpy* (Kristensen and Vinter, 2010), and data visualization, such as *Matplotlib* (Hunter, 2007), *Seaborn* (Waskom, 2021) and *ScyPy* (Virtanen et al., 2020), were also employed.

Results were mainly assessed in terms of explained variance *R* ^2^ (sklearn.metrics.explained_variance_score), main absolute percentage error (MAPE) (sklearn.metrics.mean_absolute_percentage_error), and root mean squared error (RMSE) (sklearn.metrics.mean_squared_error, squared = False). For overfitting, checking, and model validation, the same metrics were employed obtained by leave-one-out cross-validation (LOOCv) predicted data for each validation sample [sklearn.model_selection.cross_val_predict, cv = LeaveOneOut()]. Models were refined based on selection of features with non-zero coefficients where, among all initially selected features, different number of features starting from the most significant, and by steps of one, each feature was evaluated so that in the end all significant features were evaluated. For each iteration, the corresponding quality metrics were collected for further evaluation. The optimal numbers of significant features were further used in the process of model hyperparameter tuning (sklearn.model_selection.GridSearchCV). The code is available in the following Github repository: https://github.com/GustavoHBDuarte/Metabolomics_PK_proj.

## Results

### LC-MS/MS Method Validation

#### Specificity

**FIGURE 1:**
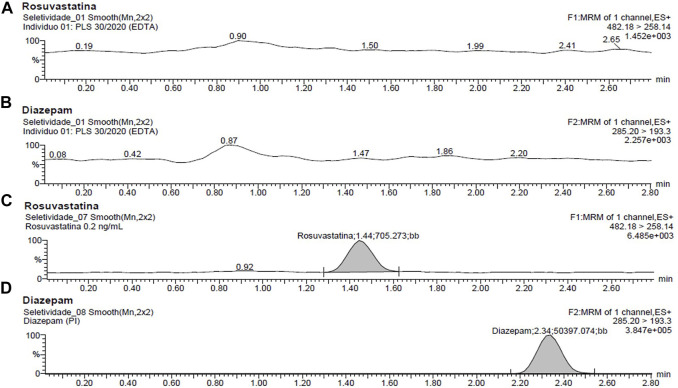
Representative multiple reaction monitoring (MRM) chromatograms of rosuvastatin in human plasma: **(A)** rosuvastatin and **(B)** diazepam blank human plasma; **(C)** rosuvastatin and **(D)** diazepam-spiked human plasma containing 0.2 ng ml^−1^ rosuvastatin and internal standard.

Specificity assessment results show that the developed method for the analysis of rosuvastatin using the selected mass transitions for MRM function was selective enough for quantitative purposes, with no apparent interferences either from endogenous compounds or from matrix effects around the analyte and IS retention times. Figure 1 portrays the chromatograms obtained from blank plasma samples and blank plasma samples spiked with rosuvastatin (0.2 ng ml^−1^) and IS.

#### Carryover

To evaluate the presence of carryover effects, three injections of blank samples were analyzed, one before and two after ULOQ. No signal of analyte was detected in any of the blank injections.

#### Matrix Effect

For matrix effect evaluation, the mean values obtained for QCs and IS were 108.4% (SD = 10.9%) and 104.4% (SD = 8.6%), respectively, demonstrating that no apparent matrix effect affects the rosuvastatin determination in human plasma samples employing the developed method.

#### Calibration Curve and LLOQ

The calibration curves were plotted as the peak area (rosuvastatin/IS)/drug concentration ratio. Linearity was achieved over the concentration range of 0.2–50 ng ml^−1^ with the correlation coefficient (*R* ^2^) greater than 0.99 for all the curves. The RE of the mean concentration measured ranged from −6.17% to 9.50%, and the RSD ranged from 3.02% to 14.46%. Table 1 portrays the overall results for linearity evaluation.

**TABLE 1:** Calibration curves from one batch of the validation section.

Nominal concentration (ng ml^−1^)	Mean concentration (ng ml^−1^)	RSD (%) (*n* = 3)	RE (%)
0.2	0.188	10.94	−6.17
1	0.921	6.66	−7.87
5	4.698	3.79	−6.05
10	10.623	14.46	6.23
20	19.950	3.02	−0.25
30	28.805	6.36	−3.98
40	40.173	6.19	0.43
50	54.749	4.02	9.50

#### Accuracy and Precision

Accuracy and precision were calculated in terms of intra- and inter-batch variation at QC levels, and the corresponding results are shown in Table 2. Intra- and inter-batch precision (RSD) ranged from 5.87 to 14.16 and 5.84 to 10.59, respectively, whereas intra- and inter-batch accuracy ranged from −0.31% to 5.83% and −3.04% to 1.88%, respectively, indicating that the method is reliable and reproducible within its analytical range.

**TABLE 2:** Precision and accuracy (analysis, spiking plasma samples at three different concentrations).

Nominal concentration (ng ml^−1^)	Intra-batch				Inter-batch			
	Mean concentration measured (ng ml^−1^)	SD	RSD (%) (*n* = 5)	Relative error (%)	Mean concentration measured (ng ml^−1^)	SD	RSD (%) (*n* = 5)	Relative error (%)
0.6	0.64	0.09	14.16	5.83	0.61	0.06	10.59	1.26
25	24.92	1.18	4.74	−0.31	24.24	1.42	5.84	−3.04
38	37.57	2.21	5.87	−1.13	38.72	2.76	7.12	1.88

#### Stability

The four-cycle freezing and thawing stability (−70°C to room temperature) evaluations have shown that the analyte is stable in human plasma at QC levels for all cycles. Additionally, no analyte degradation was detected in an autosampler tray stability assessment over a 24-h storage period, with the measured analyte values ranging from 83.17% to 111.58% of their corresponding nominal values. Moreover, for long-term evaluation, results have shown that rosuvastatin was stable over a 160-day storage period at −70°C, with measured analyte values ranging from 90.42% to 97.17% of the nominal values.

#### Application in a Pharmacokinetic Study and Statistical Evaluation of Pharmacokinetic Parameters

**FIGURE 2:**
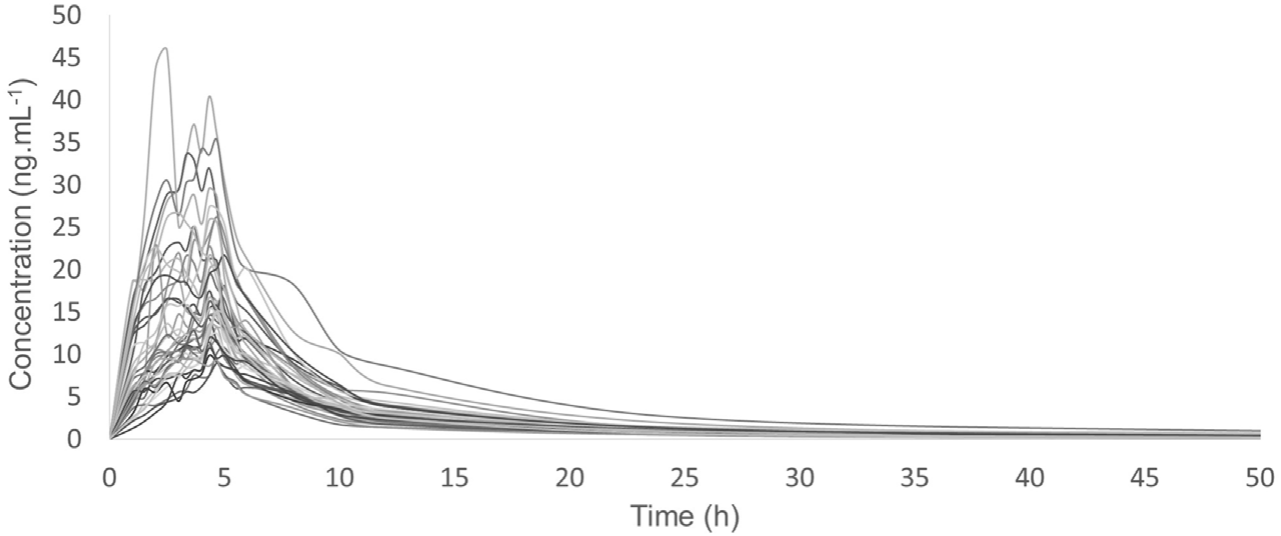
Plasma concentration–time curves of rosuvastatin reference formulation administered to 40 volunteers.

The validated analytical method was then applied to measure rosuvastatin concentration in healthy volunteers in order to obtain the main pharmacokinetic parameters. This study was conducted with 40 volunteers after a single oral dose (20 mg) of the drug. Figure 2 portrays the typical plasma concentration versus time profiles. Plasmatic concentrations of rosuvastatin ranged within the standard curve and remained above the LLOQ of 0.2 ng ml^−1^ for the entire sampling period.

The observed AUC value from time 0 to the last sampling time (AUC_0–t_) was 154.13 ± 63.06 ng h/ml, whereas the AUC from 0 to ∞ (AUC_0–∞_) was 162.85 ± 64.96 ng h/ml. The elimination half-life (*t*_1/2_) was 15.10 ± 7.91 h.

The observed *C*_max_ was 19.53 ± 8.64 ng ml^−1^, whereas the *T*_max_ was 4.18 ± 0.72 h. Moreover, the AUC_0–t_/AUC_0–∞_ mean ratio was higher than 80%.

### UPLC-QTOF-MS^E^-Based Untargeted Profiling for AUC and *C*_max_ Predictions From Predose Endogenous Plasma Metabolites

After the successful application of the validated method, the main pharmacokinetic parameters such as AUC and *C*_max_ were obtained for the rosuvastatin formulation administered to the study volunteers. As result, despite all the controlling for physiological and environmental conditions, it was possible to note a seven-fold difference between the lowest and the highest AUC values as well as an eight-fold difference between the lowest and the highest *C*_max_ values. The extent of inter-individual variation can be clearly seen in Figure 2. Thereafter, plasma predose endogenous metabolite profiling was acquired using liquid chromatography-high-resolution mass spectrometry. Typical ESI (+) and ESI (−) chromatograms for QC samples of the applied method for metabolite profiling are displayed in Supplementary Figure 1.

The corresponding acquired data was assessed to investigate the presence of metabolites that can be helpful as predictors of individual AUC and *C*_max_ variation. To do so, an Elastic Net linear regression machine learning model was built using acquired data from predose endogenous metabolite profiling as independent *X* variables and the pharmacokinetic parameters as dependent *Y* variables. Initially, four different models were built corresponding to the two different ionization modes employed to acquire data [ESI (+) and ESI (−)], each of them was used to predict their corresponding pharmacokinetic parameters (AUC or *C*_max_) using all detected molecular features to both evaluate the performance of the corresponding models and screen for the best features. Raw data processing, cleaning, and filtering of ESI (+) data resulted in 2,609 molecular features available for machine learning modeling, whereas 2,446 molecular features remained available after processing and filtering ESI (−) data.

Initial evaluation pointed to poor performance of models using all molecular features available for all models assessed, in part probably due to the large dimensional data space typically obtained by metabolite profiling experiments and the unbalanced tradeoff between the number of variables and the available number of samples. Thus, in order to improve model performance as well as to screen for helpful predictors, the most promising molecular features in the initial evaluation were selected to build further refined models, decreasing the number of variables and making data simpler and easier for Elastic Net to perform regression and data interpretation. Variables were selected on the basis of their corresponding model coefficients, excluding variables with zero coefficient values. Additionally, after initial variable selection by coefficient values, the model performances using the selected variables were evaluated by increasing the number of significant features starting from the most significant feature and adding more features, increasing by steps of one towards the least significant selected feature. Information about model metrics from each iteration was collected for both the training set and the LOOCv testing set in order to select the optimal number of significant features. Metrics resulting from the LOOCv testing set were prioritized for this selection. After this step, 69 and 27 molecular features remained for AUC and *C*_max_ models for ESI (+) datasets, respectively, whereas 49 and 17 molecular features remained for AUC and *C*_max_ models for ESI (−) datasets, respectively. It is important to mention that LOOCv was chosen as the cross-validation method due to its accurate, reliable, robust, and unbiased estimate of model performance and due to the reduced number of samples available for both model training and testing. Before running the algorithm itself for these models, data were initially normalized by the area of the internal standard in order to minimize the effect of any eventual poor injection that may have occurred. Then, using MetaboAnalyst 5.0 (Pang et al., 2021), data were also normalized using the option “Normalization by a pooled sample from group,” the group QC was selected, and data were cubic root transformed. Finally, data were scaled using the robust scaler method (sklearn.preprocessing.RobustScaler).

The next step of model refinement was to adjust algorithm hyperparameters. These parameters are applied to configure either the characteristics of the learning procedure or the structure of the underlying model. For that purpose, the exhaustive grid search method was employed. This method exhaustively generates candidates from previously specified parameter values. Then, after selecting a dataset for fitting, all combinations of parameter values are evaluated and the corresponding best combination is retained. The set of parameters and their corresponding ranges of values chosen for evaluation resulted in 4,800 experiments for each model after the tuning had been selected. The final optimized values for each dataset are shown on Supplementary Table S1 in the Supplementary Material data file.

Alternatively, we also built an Elastic Net model to predict AUC and *C*_max_ values for rosuvastatin using volunteers’ clinical features (the results from the set of laboratory tests) as *X* variables in order to further compare their performance with the corresponding model using metabolomics data as predictors and evaluate the best predictors of pharmacokinetic parameters. Finally, an integrated model using both volunteers’ clinical features and metabolomics data was also built and evaluated. The 44 applied volunteers’ clinical features are listed in the Supplementary Table 1.

#### AUC Predictions

**FIGURE 3:**
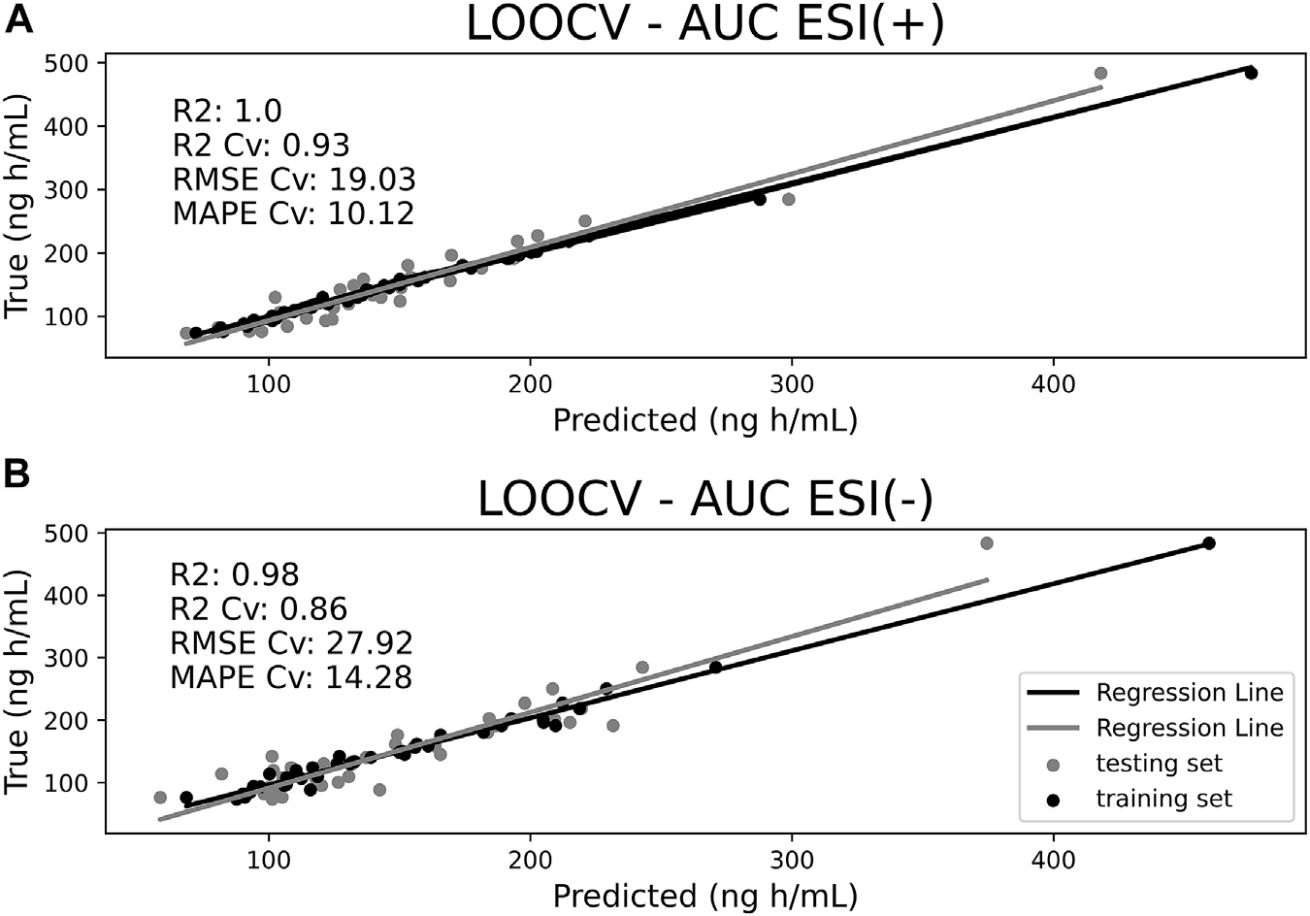
Area under curve (AUC) predictions for ESI (+) **(A)** and ESI (−) **(B)** datasets comparing training samples (black dots) with leave-one-out cross-validation (LOOCv) testing samples (gray dots). *X*-axis represents model-predicted values and *Y*-axis the true values.

The refined Elastic Net model was then applied to predict AUC values of study volunteers from predose endogenous metabolite profiling acquisition. Figure 3 portrays the predicted-against-experimental AUC values for ESI (+) and ESI (−) data where both training and LOOCv testing datasets are evaluated simultaneously. Ideally, in a good prediction model, all the samples should fall as close as possible to the regression line. The *R* ^2^ coefficient scales the extension of variance in AUC values that can be explained by the model. For ESI (+) data, *R* ^2^ achieved 1.00, whereas for ESI (−) data, *R* ^2^ achieved 0.98 for the training set. Although training *R* ^2^ results were close to 1.0, it is crucial to consider the results of the LOOCv external set in order to evaluate the model’s ability to predict unseen data so that it can be properly validated. The LOOCv *R* ^2^ Cv was 0.93 for ESI (+) and 0.86 for ESI (−). The closeness between training and Cv for *R* ^2^ indicates both models’ good ability to predict AUC values for external sets of samples.

Furthermore, root mean square error of cross-validations (RMSE Cv) and mean absolute percentage error of cross-validations (MAPE Cv) were also calculated. These parameters specify error measurements for fitted model observations. For ESI (+), RMSE Cv reached 19.03 ng h/ml and MAPE Cv reached 10.12%, while for ESI (−), RMSE Cv reached 27.92 ng h/ml and MAPE reached Cv 14.28%.

**FIGURE 4:**
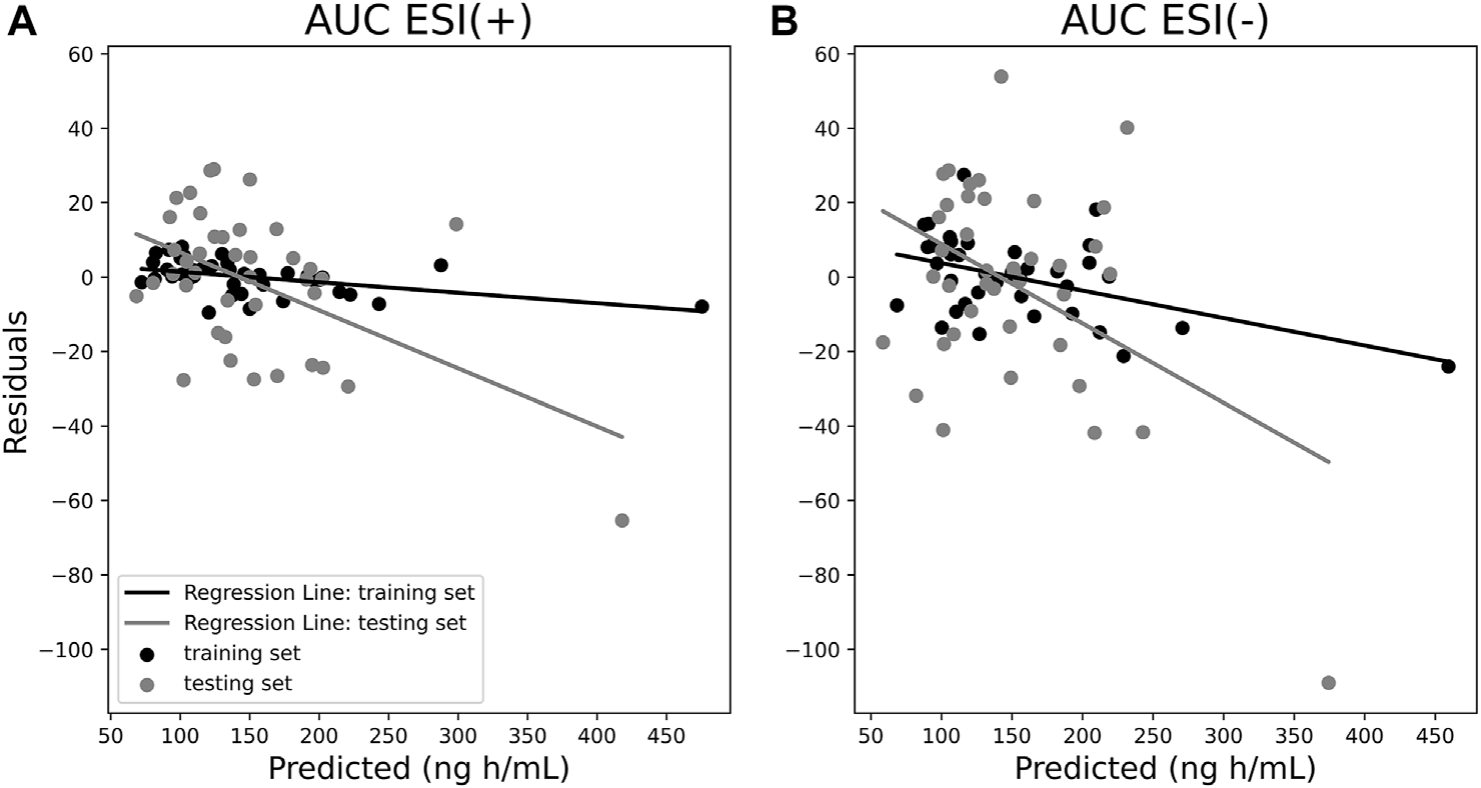
Residual plots of ESI (+) **(A)** and ESI (−) **(B)** area under curve (AUC) models for both training (black dots) and leave-one-out cross-validation (LOOCv) testing samples (gray dots) as a dispersion diagram.

From predicted and true AUC values, it is possible to obtain a residual plot. In the dispersion diagram, it is possible to note both training and LOOCv testing set samples similarly distributed around the ordinal least squares (OLS) *R* ^2^ regression line. Moreover, samples with higher AUC values had higher residuals. Considering that the frequency of observations with higher AUC values was substantially lower than the frequency of observations with lower AUC values, the selected machine learning algorithm possibly turned out to be trained differently across this AUC range, and apparently, this difference reflected on the calculated residuals. The OLS *R* ^2^ regression line value for LOOCv residuals was 0.25 for ESI (+), meaning that 25% of the variation in AUC-predicted values is explained by variation in residuals shown in the dispersion diagram, whereas for ESI (−), the OLS *R* ^2^ achieved 0.19. Furthermore, shared *Y*-axis reveals higher residuals for the ESI (−) model compared to the ESI (+) model. Figure 4 portrays the residual evaluation in the form of a dispersion diagram.

**FIGURE 5:**
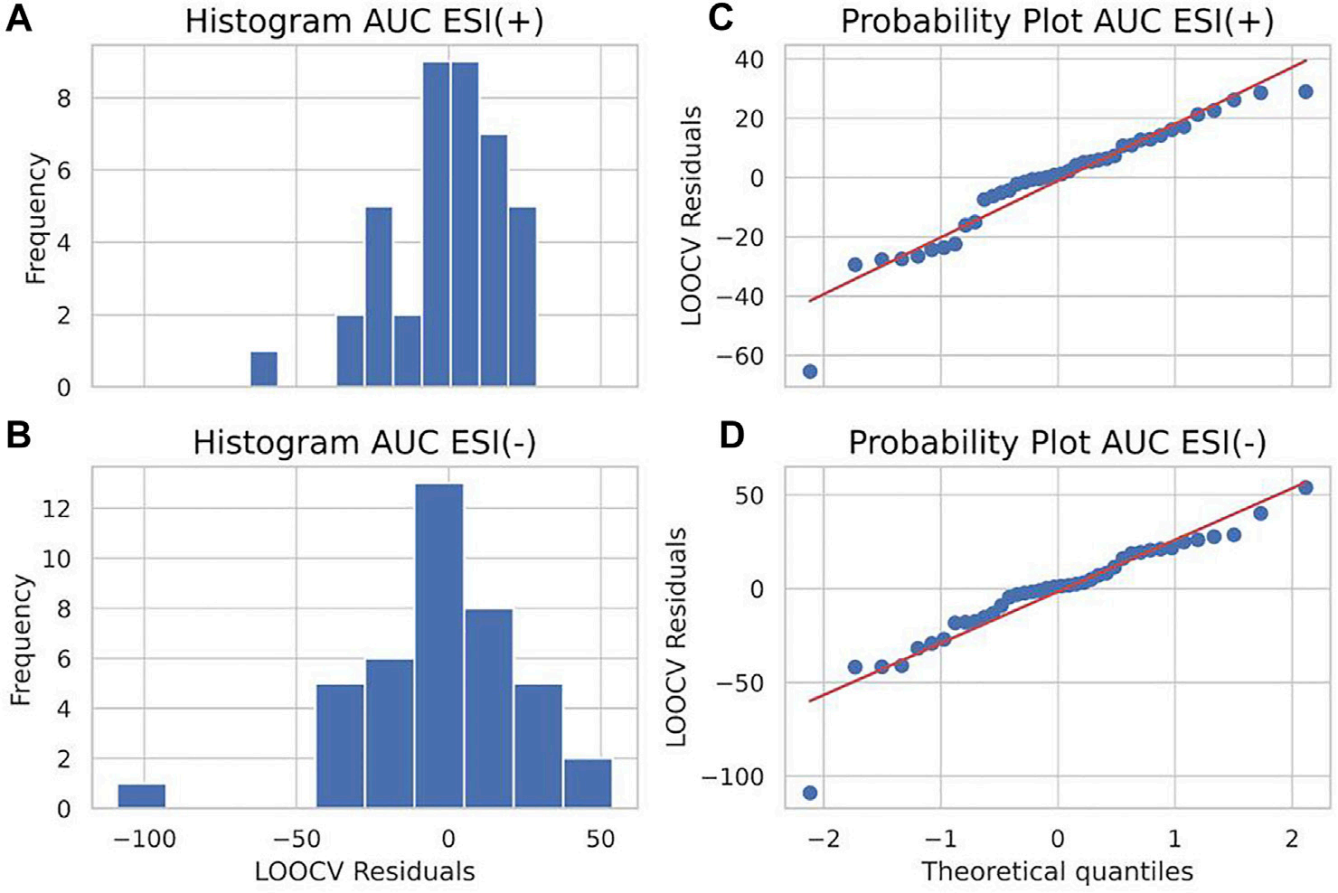
A histogram of residuals (left panels) of ESI (+) **(A)** and ESI (−) **(B)** data and probability plot of residuals (right panels) of ESI (+) **(C)** and ESI (−) **(D)** data for area under curve (AUC) predictions.

Similarly, the information provided by residuals was used to generate both histograms and probability plots to evaluate the distribution of frequencies for residuals and obtain a more in-depth visualization. Figure 5 portrays the results. A histogram inspection suggests residuals around zero as being the most frequent values for both the ESI (+) and ESI (−) models, whereas the probability plot reveals that, although not falling straight to the normality Gaussian fit line, the distribution of residuals against theoretical quantiles follows a similar trend for both ESI (+) and ESI (−), showing the consistency of the obtained data.

**FIGURE 6:**
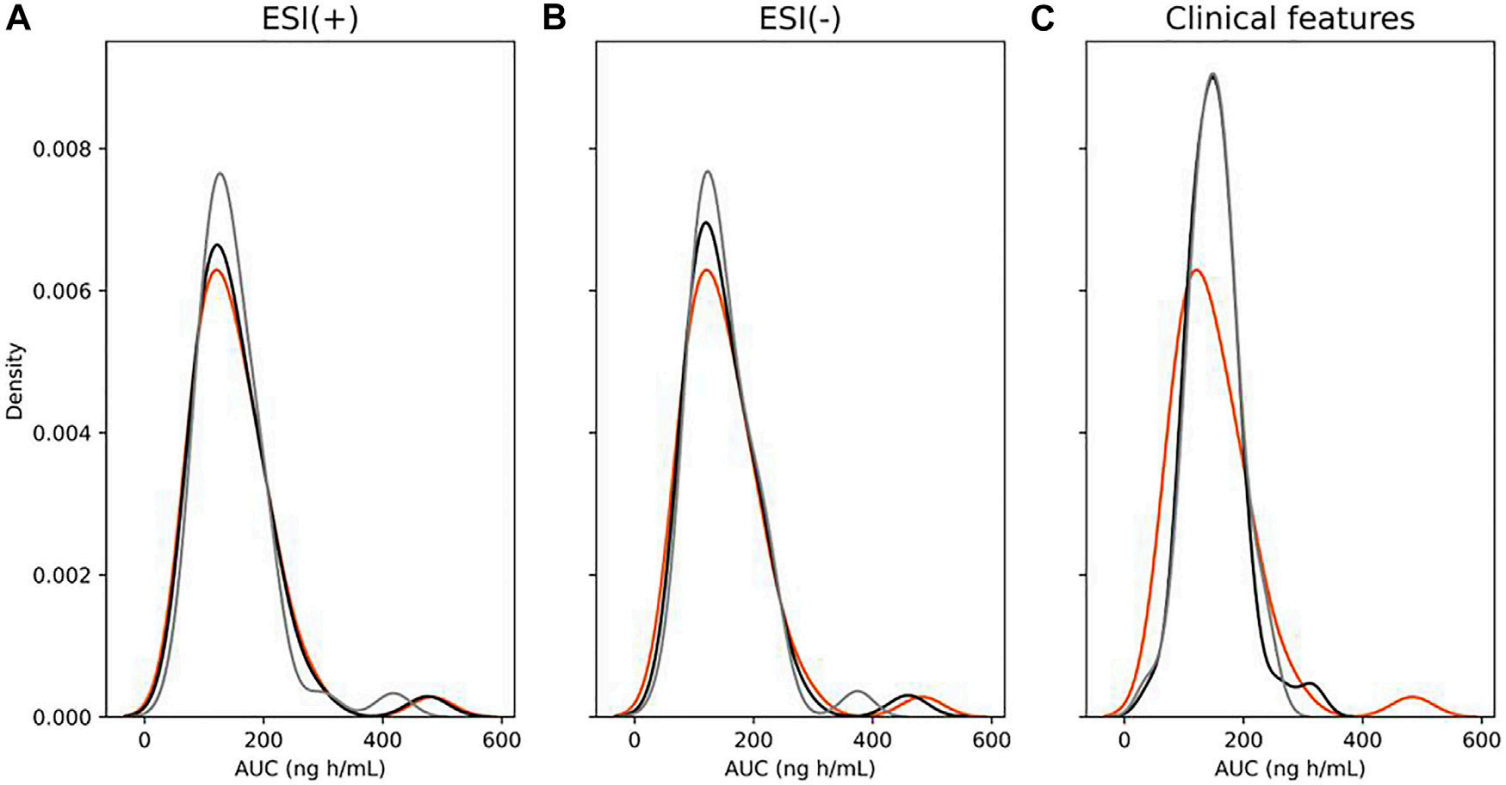
Distribution plots showing the frequency of area under curve (AUC) values of true (orange), training set (black) predicted, and leave-one-out cross-validation (LOOCv) testing (gray) predicted from ESI (+) **(A)**, ESI (−) **(B)**, and clinical features **(C)** models.

Regarding machine learning modeling, applying exclusively clinical features, unlike the performance of Elastic Net model using metabolomics data as predictors, it was not possible to achieve similar results using volunteers’ clinical features as pharmacokinetic parameter predictors for AUC. RMSE Cv reached 63.92 ng h/ml, whereas MAPE Cv reached 33.00%. The LOOCv *R* ^2^ coefficient was 0.24. Predicted AUC values from the ESI (+), ESI (−), and volunteers’ clinical feature modeling were also compared to each other from the perspective of the frequencies of their corresponding predicted values. Figure 6 demonstrates a clear representation of the extension of the lesser predictability of the clinical feature model compared to the ESI (+) and ESI (−) models by using frequency plots. Results demonstrate that the clinical feature model generates an evidently higher difference in the distance of both training and LOOCv testing frequencies of predicted values from true values compared to the same measure of both the ESI (+) and ESI (−) models, showing its considerably lower predictive ability compared to information provided by metabolomics.

The final evaluation of AUC modeling included an integrated model applying both metabolomics [ESI (+) and ESI (−)] and clinical features. The information provided by the combination of both sources did not substantially improve the performance of predictions for ESI (+). Instead, for ESI (−), a slight decrease was observed in the quality metrics of the model. LOOCv *R* ^2^ reached 0.94 and RMSE Cv and MAPE Cv reached 18.32 ng h/ml and 10.21%, respectively, for ESI (+), whereas LOOCv *R* ^2^ reached 0.85 and RMSE Cv and MAPE Cv reached 28.21 ng h/ml and 14.39%, respectively, for ESI (−).

#### *C*_max_ Predictions

**FIGURE 7:**
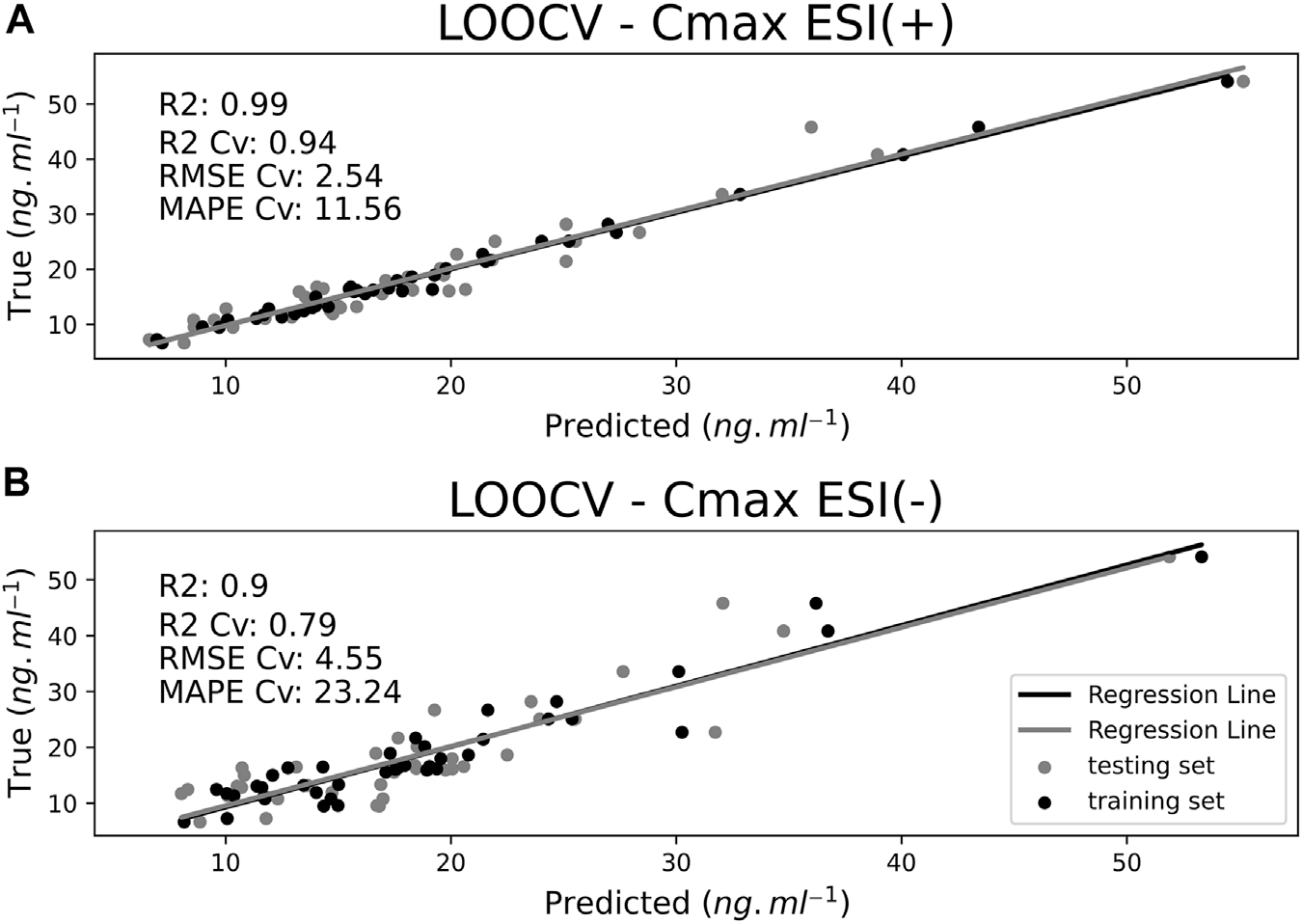
Maximum plasma drug concentration (*C*_max_) predictions for ESI (+) **(A)** and ESI (−) **(B)** datasets comparing training samples (black dots) with leave-one-out cross-validation (LOOCv) testing samples (gray dots). *X*-axis represents model-predicted values and *Y*-axis the true values.

Refined ESI (+) and ESI (−) datasets were also employed to predict *C*_max_ values of the same study volunteers from predose endogenous metabolite profiling experiments, and their corresponding performance was assessed using the same metrics used for AUC predictions. The same trend observed for AUC predictions, whereby the ESI (+) model performed better than the ESI (−) model, was also observed for *C*_max_ models. Figure 7 portrays predicted-against-experimental *C*_max_ values for ESI (+) and ESI (−) for each observation. For ESI (+), training *R* ^2^ achieved 0.99 and LOOCv testing *R* ^2^ was 0.94, whereas for ESI (−), training *R* ^2^ achieved 0.9 and LOOCv testing *R* ^2^ achieved 0.79. RMSE Cv and MAPE Cv for ESI (+) were 2.54 ng ml^−1^ and 11.56%, respectively, whereas ESI (−) RMSE Cv and MAPE Cv were 4.55 ng ml^−1^ and 23.24%, respectively.

**FIGURE 8:**
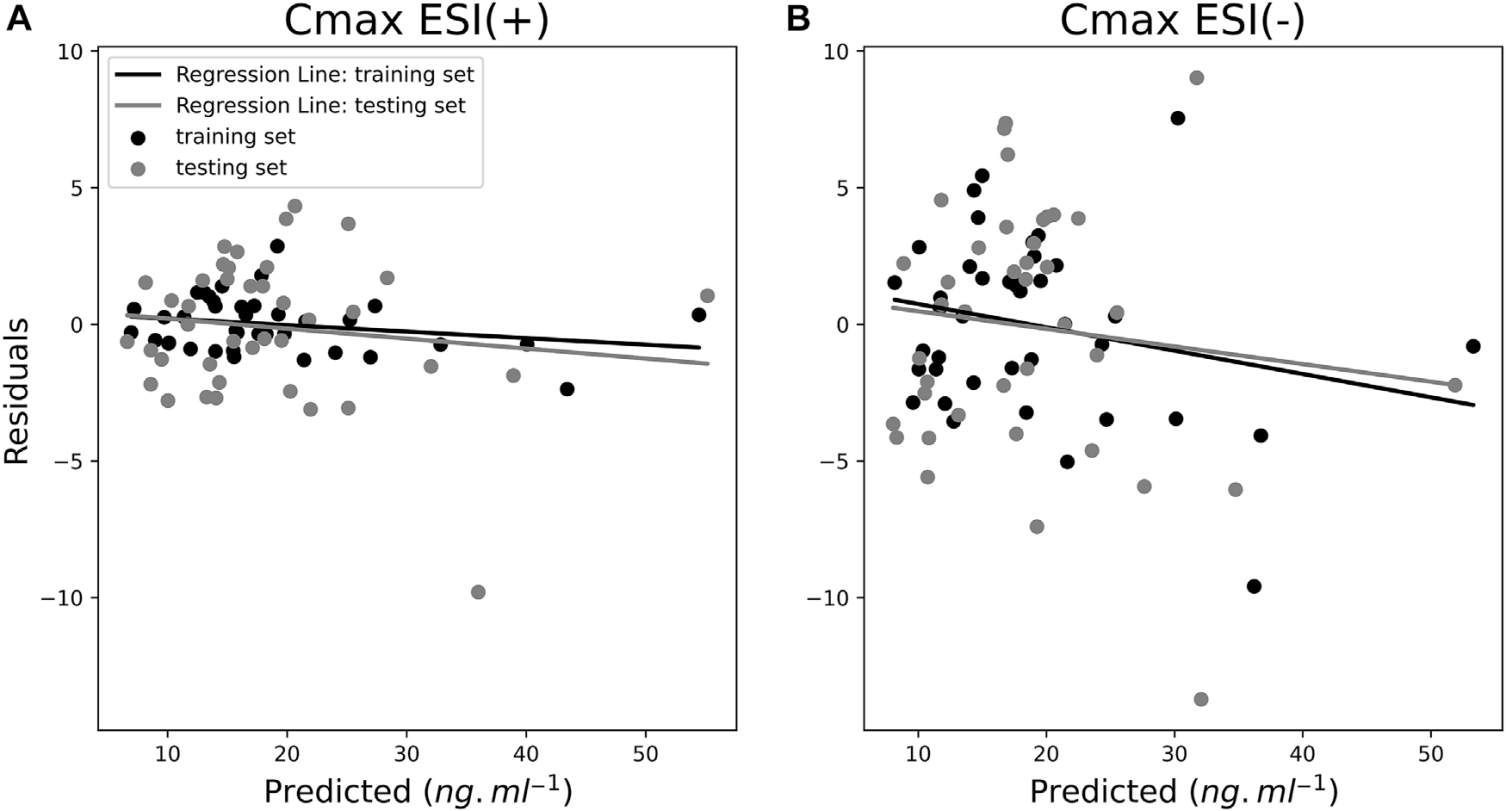
Residual plots of ESI (+) **(A)** and ESI (−) **(B)** maximum plasma drug concentration (*C*_max_) models for both training (black dots) with leave-one-out cross-validation (LOOCv) testing samples (gray dots) as dispersion diagrams.

**FIGURE 9:**
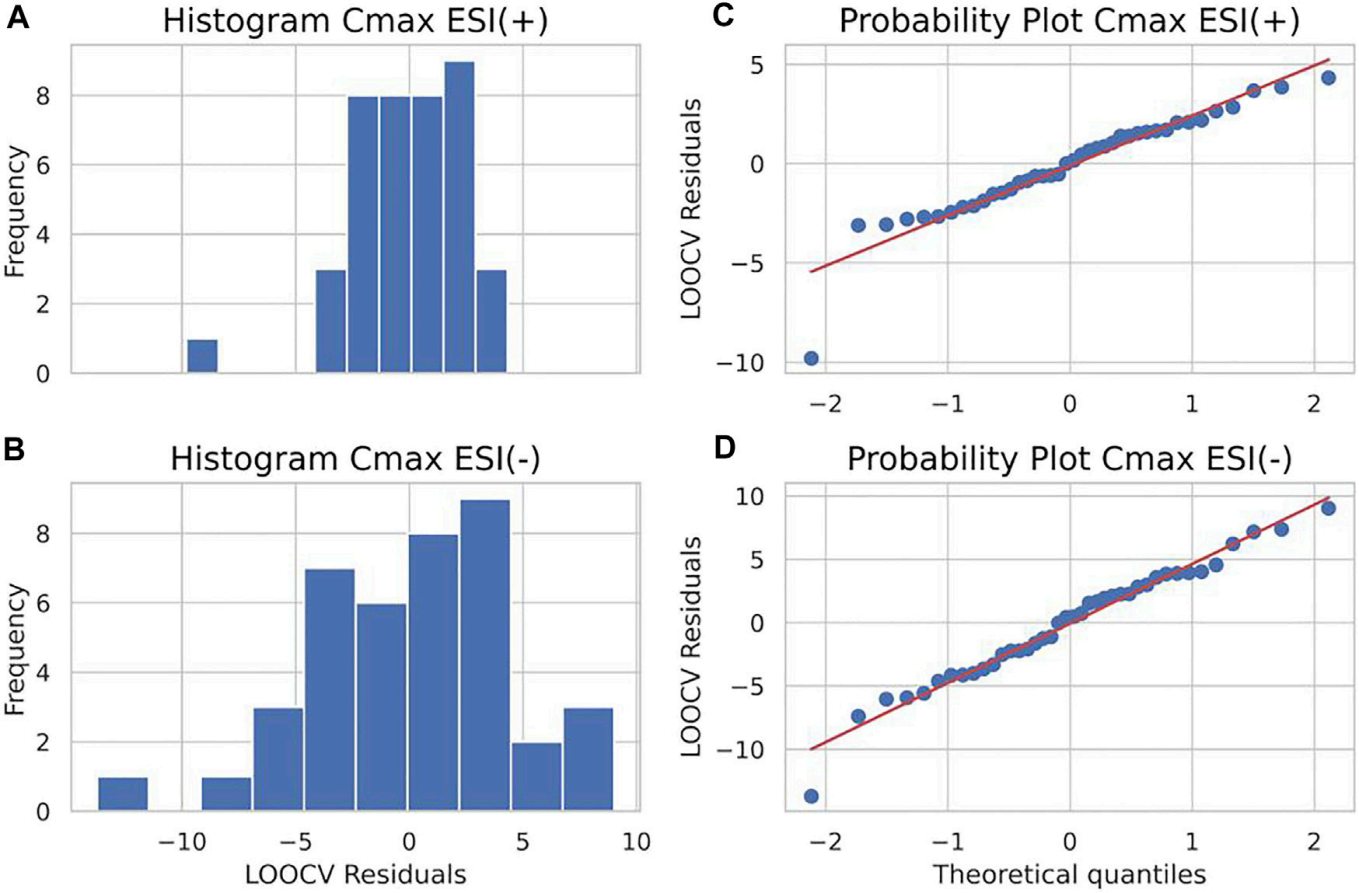
A histogram of residuals (left panels) of ESI (+) **(A)** and ESI (−) **(B)** data and probability plot of residuals (right panels) of ESI (+) **(C)** and ESI (−) **(D)** data for maximum plasma drug concentration (*C*_max_) predictions.

Figure 8 displays the residual analysis for *C*_max_ predictions as a dispersion diagram. The same trend seen for AUC predictions is also seen for *C*_max_ predictions in the corresponding plot where residuals for the ESI (−) model were higher compared to the ESI (+) ones. Although it can be observed that unlike the AUC predictions, there was a sample with a higher residual value [close to −10 in the ESI (−) model]. The sample in question was not the sample with the highest *C*_max_ value. For ESI (+), the samples with higher residuals were not necessarily the samples with higher *C*_max_ values either. The observations with higher *C*_max_ values were also less frequent than with the AUC model. The fact that, for the *C*_max_ model, the range of numerical values to predict is lower and samples are closer to each other could allow the *C*_max_ model to train better than the AUC model for predicting the numerical value of samples and spreading the residuals more evenly throughout the samples. Besides, the OLS *R* ^2^ regression line value for LOOCv residuals shown in the dispersion diagram was 0.02 for ESI (+) and 0.01 for ESI (−). Furthermore, for the distribution analysis of residuals, shown in Figure 9, the residuals with values close to zero were mainly the most frequent values. The shared *X*-axis of histograms also allowed for the visualization of the lesser spreading of residuals of the ESI (+) model compared to the ESI (−). Similarly, the shared *X*-axis of the probability plots shows, as in the AUC models, the similar distribution of data regarding residuals against theoretical quantiles, regardless of ionization mode, confirming the consistency of obtained data as the data points apparently seem to fall closer to the Gaussian regression line compared to the AUC model and, therefore, a more normal-like distribution of residuals around zero.

**FIGURE 10:**
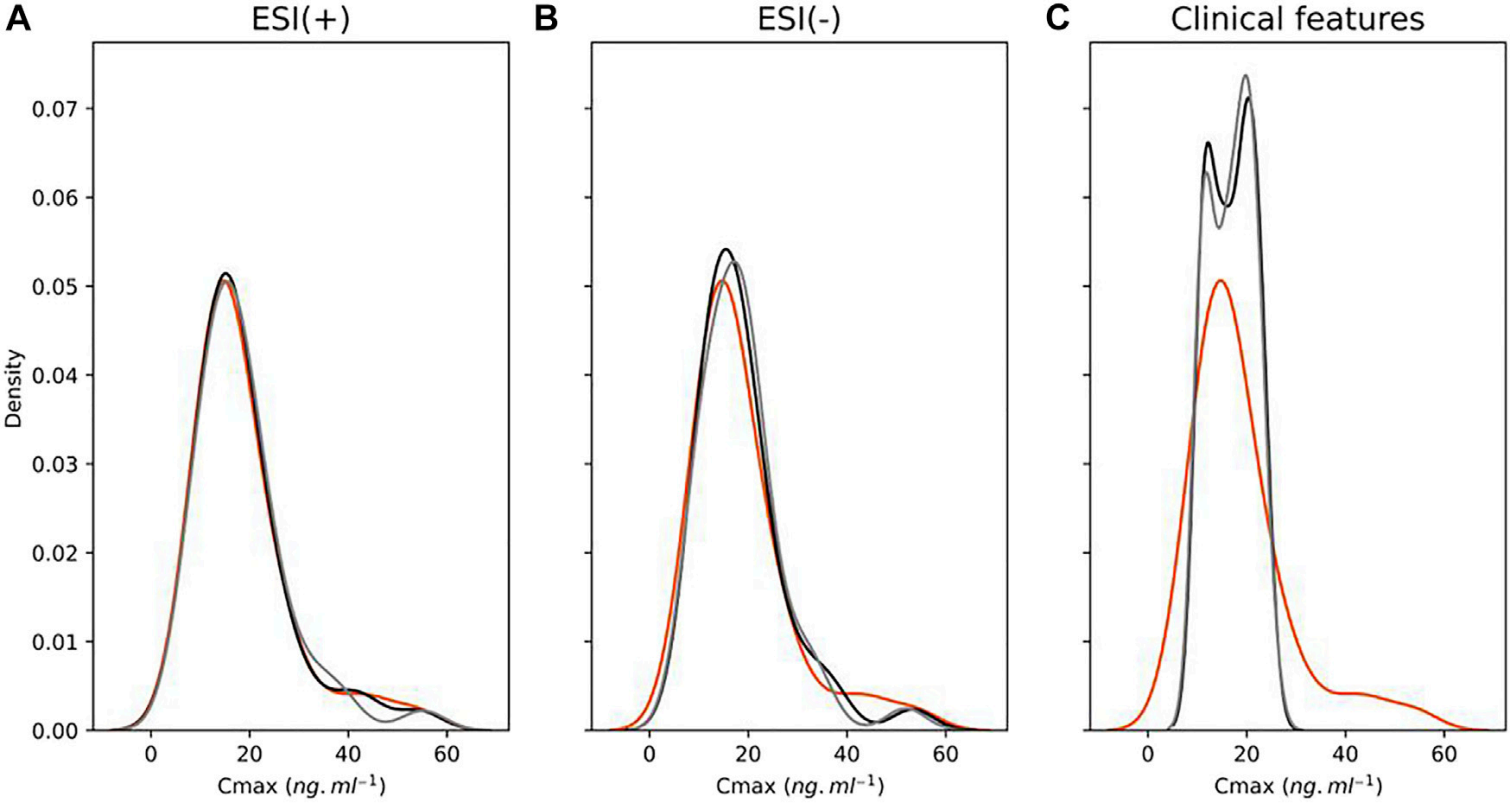
Distribution plots showing the frequency of maximum plasma drug concentration (*C*_max_) values of true (orange), training set (black) predicted, and leave-one-out cross-validation (LOOCv) testing (gray) predicted from ESI (+) **(A)**, ESI (−) **(B)**, and clinical features **(C)** models.

Regarding *C*_max_ predictions using volunteer’s clinical features, a similar trend observed for AUC models was also seen for *C*_max_ modeling with poorer prediction ability when comparing to the model applying only metabolomics data. The LOOCv *R* ^2^ coefficient reached 0.08, whereas RMSE Cv and MAPE Cv reached 9.77 ng ml^−1^ and 34.77%, respectively, showing substantially lower correspondence between clinical features and *C*_max_ values. Figure 10 shows a similar trend for AUC predictions using volunteer’s clinical features, where predictions for both training and LOOCv testing demonstrated a considerably lower ability to predict pharmacokinetic values with a clear distortion in the frequencies of predictions for both training and LOOCv testing models. A more detailed visualization in the distribution plot of predictions for the *C*_max_ ESI (+) model reveals a closer overlapping between the frequencies of training and LOOCv testing predictions compared to the frequencies of the *C*_max_ ESI (−) and even closer if compared to the AUC ESI (+) and AUC ESI (−) training and LOOCv predictions. Though there is no apparent overfitting for any of these models, this described observation is consistent with the lower distance between *R* ^2^ and LOOCv *R* ^2^ for the *C*_max_ ESI (+) model compared to the same measurement of the *C*_max_ ESI (−), AUC ESI (+), and AUC ESI (−) models.

In addition, regarding the integrated model, the same behavior observed for AUC predictions was observed for *C*_max_ predictions with the application of both clinical and metabolomic information in the same dataset not providing significant improvements over the model using only metabolomic information. Supplementary Table S2 in the Supplementary Material data file provides an overall visualization of the performance metrics for all the evaluations carried out in this work.

## Discussion

The metabolomic approach used in this work was based on the hypothesis that the metabolic profile of plasma may contain molecular information useful for predicting the pharmacokinetic response of a drug even if no genetic information is known at first. Thus, we used the plasma predose endogenous metabolite profiling of volunteers in predose, acquired from the UPLC-QTOF-MS^E^ platform, to build the Elastic Net linear regression machine learning model for prediction of the pharmacokinetic parameters of AUC and *C*_max_. Although profiles were acquired in both ESI (−) and ESI (+) acquisition modes, the latter was the one that provided the best models according to MAPE and RMSE errors. Likewise, it is important to add that, in isolation, no single metabolite was able to present a good correlation with *C*_max_ and AUC values, only when they were evaluated together.

From these models, we were able to prepare a list (Table 3) of 16 compounds most relevant for the prediction of the pharmacokinetic values and for which it was possible to suggest at least one chemical identity. Identification suggestions were made automatically by the Progenesis QI software based on parameters such as the following: mass error, that is, the difference, in parts per million (ppm), between the measured molecular mass and the theoretical mass for the proposed molecular formula; isotopic similarity between the experimental and theoretical spectra; and the match between fragments of theoretical compared to experimental masses (Mecatti et al., 2020). The table also includes the codes (identifiers) that link the suggested identities to public databases of metabolites and the coefficients of these features in the prediction models (see Supplementary Tables 2 and 3 for a complete list of molecular features that contributed to the prediction models and their respective coefficients). A pathway analysis diagram was built with the MetaboAnalyst 5.0 free platform (Supplementary Figure S2 in the Supplementary Material data file).

**TABLE 3:** Most relevant and putatively identified metabolites selected by the Elastic Net regression model for pharmacokinetic parameters prediction.

Feature code^a^ (RT_*m/z*)	IM^b^	Molecular formula	Putative assignment	Mass error (ppm)	Diagnostic fragments (*m*/*z*)^c^	Identifiers^d^	Coefficient^e^ (AUC/*C*_max_)
Sterols and bile acids							
8.31_329.2476 *m*/*z*	−	C22H36O3	Dinorlithocholic acid	−3.02	319.264	LMST04050010	1.1/0.1*
6.45_403.2840 *m*/*z*	−	C25H42O5	Homoavicholic acid	−3.32	277.217	LMST04070041	1.6/0.1*
7.36_452.3121 *m*/*z*	+	C8H11N3O3	1α-hydroxy-21-nor-20-oxavitamin D3	+3.92	131.049 110.041 107.050 79.039	LMST03020026	1.1/0.0*
6.45_447.2739 *m*/*z*	−	C25H38O4	3-oxochenodeoxycholic acid	−3.27	403.285 227.217	HMDB0000541	2.1/0.1*
Carboxylated metabolites							
0.33_180.0628 *n*	−	C6H12O6	Fuconic acid	−3.51	225.062 179.0561 161.046	LMFA01050532	−2.3/−0.9*
4.31_439.1598 *m*/*z*	−	C8H11N3O3	N-acetylhistidine	+3.92	NA	C02997	−2.0/−07*
0.36_217.0297 *m*/*z*	−	C7H12N2O5S	Cysteinyl aspartate	+3.41	167.021 166.015	HMDB0028771	−2.0/−0.3*
0.38_97.0286 *m*/*z*	+	C5H8O4	Glutaric acid	−3.73	85.029	C00489	−0.8/−0.1*
Lipids							
6.60_796.4764 *m*/*z*	−	C42H72NO11P	OxPS 36:5+1O	−0.76	255.232	LipidBlast458932	1.3/0.1*
6.20_446.2288 *m*/*z*	−	C19H40NO7P	LysoPE(14:0)	−0.18	415.211 413.915 245.990	HMDB0011470	1.7/0.0*
0.58_1191.1731 *m*/*z*	−	C80H155NO5	Cer-EODS d80:2/Cer-EOS d80:2	+0.31	–	LipidBlast197294	−1.7/−0.3*
10.22_529.4246 *m*/*z*	−	C34H60O5	DG(31:3)	−3.03	293.212 271.228	LMGL02010362	1.2/0.2*
0.31_830.7578 *m*/*z*	−	C52H99NO3	Cer-NS d52:3^d^	−3.63	NA	LipidBlast013435	−1.3/−0.4
6.86_1547.9838 *m*/*z*	−	C38H73NO11S	3-O-Sulfogalactosylceramide (32:1)	+3.18	266.233	C06125	1.6/0.0*
3.26_916.5829 *m*/*z*	+	C51H81O8P	PA 40:8	−0.31	NA	LipidBlast460837	4.3/0.7*

a RT, retention time; *m/z*, mass-to-charge ratio.

b IM, ionization mode.

c Fragments that are matching between experimental data and database.

d Identifiers: LMXX, metabolites described in the LipidMaps (https://www.lipidmaps.org/data/structure/); HMDBXXXXXXX, metabolites described in the Human Metabolome Database (HMDB—https://hmdb.ca/); CXXXXX, described in the Kyoto Encyclopedia of Genes and Genomes database (KEGG—https://www.genome.jp/kegg/); and LipidBlastXXXXX, described in the MassBank of North America (MoNA—https://mona.fiehnlab.ucdavis.edu/).

e Coefficients in the refined AUC and *C*_max_ models (in that order). Coefficients highlighted with an asterisk (*) corresponds to initial model.

NA, not applicable. All listed compounds met Metabolomics Standards Initiative (MSI) level 2 identification requirements, except for b, d, i, and m, which met level 3 requirements (Schrimpe-Rutledge et al., 2016).

Metabolites were grouped into four groups: steroids and bile acids, carboxylates, lipids, and salts. The key factors to understand the potential role of these metabolites for the prediction of rosuvastatin pharmacokinetic parameters rely on their potential role in the transport mechanism of this substance. The pharmacokinetic parameter of *C*_max_ is related to the variability in gastrointestinal transport. AUC, in turn, reflects the variability in the extent of absorption and elimination and may thus involve hepatic clearance. These processes involve the participation of transporters, which has been implicated as one of the factors responsible for the variation in the absorption, distribution, metabolism, and excretion (ADME) of several drugs (Soars et al., 2009; Harwood et al., 2013). Interactions between these transporters and circulating metabolites may underlie how this predose metabolic information can influence the pharmacokinetic parameters of rosuvastatin (Luvai et al., 2012).

Rosuvastatin, among other statins, is transported by the OATP1B1, an influx transporter expressed in the basolateral membrane of human hepatocytes and encoded by the *SLCO1B1* gene (Hua et al., 2012). The relationship between this transporter and the pharmacokinetics of statins is so relevant that it has been established that *SLCO1B1* is one of the risk predictors of myopathy caused by statins (Kisor et al., 2019). Specifically for rosuvastatin, several studies show that *SLCO1B1* c.521CC, a single-nucleotide polymorphism capable of decreasing the transporter activity, causes an increase in plasma concentrations of rosuvastatin (Pasanen et al., 2007; Choi et al., 2008; Lee et al., 2013; Birmingham et al., 2015a; Birmingham et al., 2015b; Wan et al., 2015; Liu et al., 2016; Kim et al., 2019). This transporter also acts on many other substrates, including bile salts, hormones, and steroid conjugates (Ho and Kim, 2005; DeGorter et al., 2012). Genetic variants of *SLCO1B1*, including c.521CC, are associated with an increase in plasma concentrations of bile acids (Xiang et al., 2009).

Given these data, it has been suggested that competitive interactions with this transporter may contribute to a decrease in the hepatic absorption of statins, impacting the pharmacokinetic parameters of these drugs. It has been reported that intravenous administration of rifampicin, an inhibitor of the hepatic transporter OATP1B1, substantially increased the plasma concentrations of atorvastatin and its metabolites (Lau et al., 2007). The same can occur with respect to circulating endogenous metabolites. It has been described that pretreatment concentrations of some primary and secondary bile acids were correlated with the response to simvastatin treatment (Krauss et al., 2013), suggesting a direct relationship between the presence of these basal metabolites and the drug’s bioavailability. Similarly, cholesteryl ester predose levels were well correlated with the simvastatin response (Kaddurah-Daouk et al., 2010; Krauss et al., 2013). It was also demonstrated that free cholesterol was among the metabolites detected in the predose of individuals to whom a single dose of atorvastatin was administered, and there was a correlation between *C*_max_ and AUC and the basal levels of this metabolite (Huang et al., 2015). Aligned with these reports, the bile acids dinorlithocholic, homoavicholic, and 3-oxochenodeoxycholic acids and the steroidal lipid 1β-hydroxy-21-nor-20-oxavitamin D3 are among the listed metabolites that most contributed to the prediction of pharmacokinetic parameters, especially AUC. We observed that the positive values of the coefficients in the model’s features point to a direct association, that is, the higher the plasma levels of those metabolites, the greater the competition for OATP1B1 transporters, the lower the liver transport, and the slower the hepatic abortion of rosuvastatin. It is important to mention that, as specified before, other transporters influence the ADME of rosuvastatin, in particular, *ABCG2*, which may present polymorphisms that can be of special relevance for some populations (Lee et al., 2013). However, predose metabolites that could potentially influence these transporters were not accessed in this study.

**FIGURE 11:**
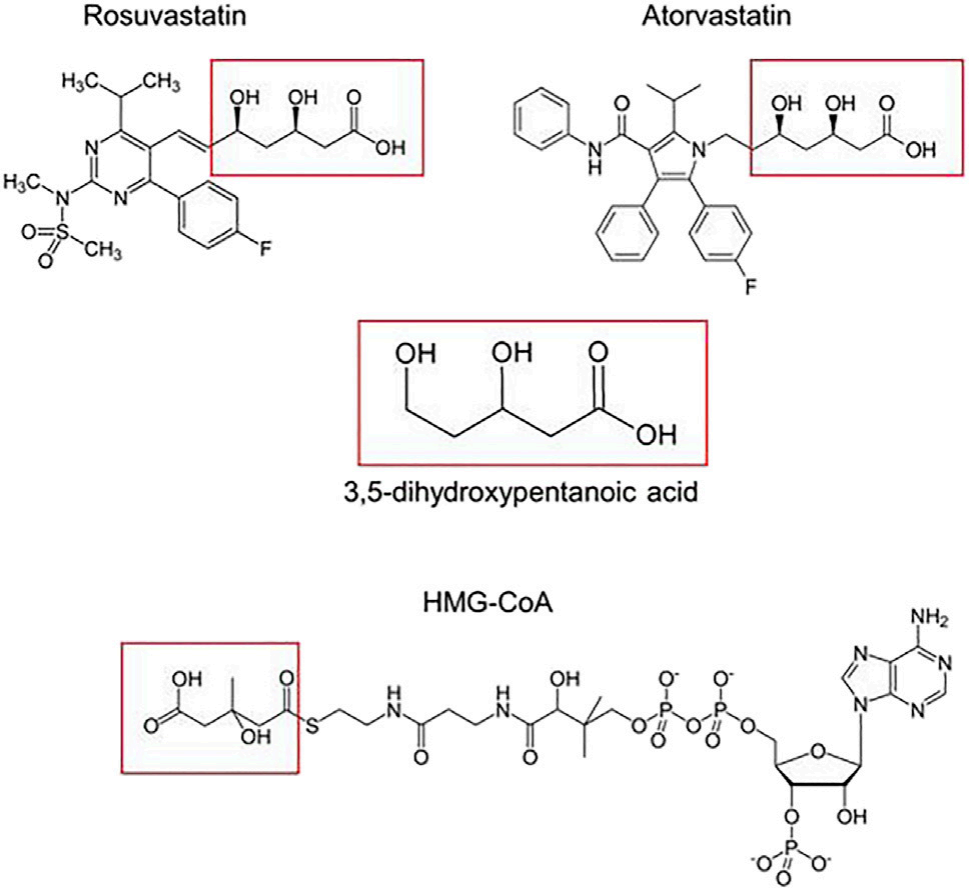
Pharmacophore group of statins. The 3,5-dihydroxypentanoic acid is the common core among all statins and is responsible for inhibiting the enzyme 3-hydroxy-3-methylglutaryl–coenzyme A reductase (HMGR). These monocarboxylated portions (highlighted in red) mimic the anchorage site of 3-hydroxy-3-methylglutaryl-coenzyme A (HMG-CoA) in the reductase enzyme, during the reductive deacylation step in cholesterol synthesis.

The pharmacophoric group of statins is the 3,5-dihydroxypentanoic acid monocarboxylated nucleus (Figure 11). Whether in the closed configuration of lovastatin and simvastatin or in the open configuration of atorvastatin and rosuvastatin, when this group is anchored in the enzyme HMGR (Istvan and Deisenhofer, 2001), they become unavailable for binding with HMG-CoA, which blocks a key step in cholesterol biosynthesis. Additionally, the transport of monocarboxylate compounds in the gastrointestinal tract has been demonstrated to be catalyzed by the proton-linked monocarboxylate 10 transporters (MCT 10). Previous studies have shown that atorvastatin is transported by monocarboxylate transporters (*MCTs*) (Wu et al., 2000; Goto et al., 2005). Thus, an MCT-mediated inhibition of intestinal absorption of rosuvastatin can substantially decrease the bioavailability of the drug. The negative values of the coefficients of the monocarboxylic compounds fuconic acid, n-acetylhistidine, cysteinyl aspartate, and glutaric acid in Table 3, the latter three notable amino acids, demonstrate the negative correlation between these metabolites and the pharmacokinetic parameters of rosuvastatin. The potential effects that these metabolites exert on rosuvastatin pharmacokinetics may occur through competitive interactions with MCT intestinal transporters. In agreement with our observations, it has previously been shown that lower levels of the monocarboxylated amino acids 2-hydroxybutyric acid, tryptophan, tyrosine, phenylalanine, leucine, isoleucine, and others were correlated with higher values of *C*_max_ and AUC (Huang et al., 2015). Likewise, lower baseline levels of 2-hydroxyvaleric, succinic, and stearic acids demonstrated a significant correlation with the decrease in low-density cholesterol caused by simvastatin (Trupp et al., 2012).

Regarding the lipids described in Table 3, we highlight lysophosphatidylethanolamine (14:0), which is consistent with the observation that the basal concentration of phosphoethanolamines was positively related to the response to treatment with simvastatin. Likewise, diacylglycerols, such as DG(31:3), demonstrated a strong correlation, both positive and negative, with the response to simvastatin (Kaddurah-Daouk et al., 2010).

In light of the results herein presented, the designed experiment has demonstrated the potential of the applied strategy in finding associations between endogenous metabolites and pharmacokinetic parameters useful in predicting inter-individual variability in drug absorption, hopefully assisting in driving personalized drug therapy strategies, with the aim of reducing adverse effects and maximizing efficacy. In fact, since the anticholesterolemic activity of statins fundamentally occurs in the liver, the systemic presence of these molecules is potentially more correlated with the myotoxic effects of this class of drugs than with therapeutic ones. Thus, an individual predose prediction of plasma *C*_max_ and AUC values would find relevant clinical application in potentially predicting which individuals would be more susceptible to presenting undesirable side effects. Data acquisition and processing strategies were able to reveal valuable information initially hidden in the huge amount of data typically provided by untargeted metabolomics experiments. Complementally, the rich set of findings of the refined approach also include some putatively identified main key metabolites belonging to classes that play important roles in plasmatic concentrations of rosuvastatin and could potentially be associated with inter-individual variability in drug response. The overall results achieved in this work clearly indicate the valuable information carried by endogenous metabolites and the vast research that can be explored in this field.

## Data Availability

The datasets generated by this study can be found in the following Metabolights repository: www.ebi.ac.uk/metabolights/MTBLS3128.
