# Supplementary material for: Serum Predose Metabolic Profiling for Prediction of Rosuvastatin Pharmacokinetic Parameters in Healthy Volunteers
Source: Front Pharmacol. 2021 Nov 12;12:752960. doi: 10.3389/fphar.2021.752960 (PMC8633954; doi:10.3389/fphar.2021.752960)
Supplement: Supplementary file 3 [file DataSheet1.PDF]

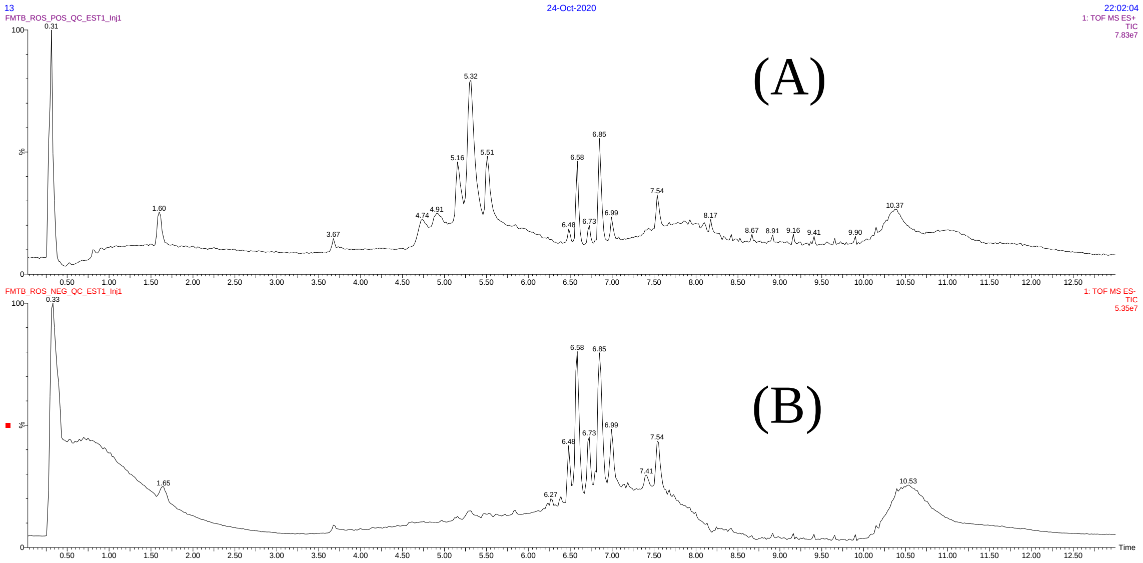

Figure S1. Typical ESI (+) **(A)** and ESI(-) **(B)** chromatograms for QC samples of the applied method for metabolite profiling.

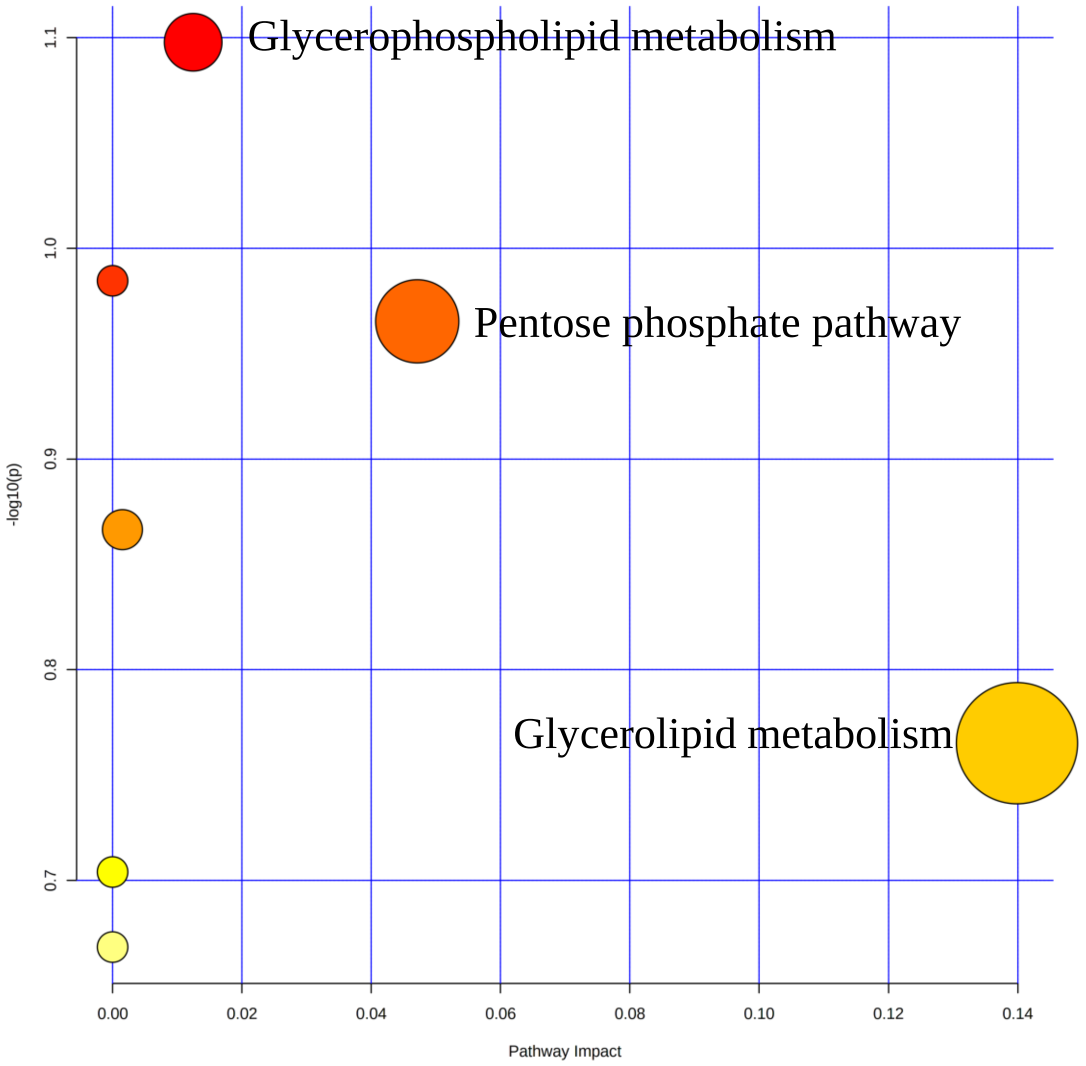

Figure S2. Metaboanalyst (http://www.metaboanalyst.ca) generated Pathway Analysis describing the impact of significant selected metabolites. The Y-axis is the original P values obtained from the pathway analysis, and the X-axis is the influence value of pathways obtained from the topological analysis.

Table S1 Overall visualization of the optimized hyperparameters for all the evaluations carried out.

dataset applied	Ionization mode	Parameter	alpha	fit_intercept	l1_ratio	max_iter	precompute	selection	warm_start
Metabolomics	ESI (+)	Cmax	0.1	True	0.1	1000	False	cyclic	True
		AUC	0.3	True	0.3	10000	False	random	False
	ESI (-)	Cmax	0.2	True	0.1	100000	True	random	True
		AUC	0.4	True	0.1	100000	True	random	True
Clinical features	-	Cmax	0.9	True	1.0	1000	False	random	True
	-	AUC	0.1	True	0.8	10000	False	random	False
Integrated model	ESI (+)	Cmax	0.2	True	0.1	10000	False	random	False
		AUC	0.2	True	0.1	1000	True	cyclic	True
	ESI (-)	Cmax	0.2	True	0.1	10000	True	random	False
		AUC	0.5	True	0.1	1000	True	random	True

Table S2 Overall visualization of the performance metrics for all the evaluations carried out.

dataset applied	Ionization mode	Pharmaco kinetic parameter	Trainning set			LOOCv testing set		
			R2	MAPE	RMSE	R2	MAPE	RMSE
Metabolomics	ESI (+)	C_{max}	0.99	4.77	0.96	0.94	11.56	2.54
		AUC	1.00	2.56	4.31	0.93	10.12	19.03
	ESI (-)	C_{max}	0.90	2.56	3.17	0.79	23.24	4.55
		AUC	0.98	6.28	10.55	0.86	14.28	27.92
Clinical features	-	C_{max}	0.21	31.96	9.05	0.07	34.77	9.78
	-	AUC	0.60	25.72	46.53	0.24	33.00	63.92
Integrated model	ESI (+)	C_{max}	0.84	5.23	1.08	0.93	11.44	2.69
		AUC	1.00	2.05	3.38	0.94	10.22	18.32
	ESI (-)	C_{max}	0.90	15.66	3.17	0.79	23.25	4.55
		AUC	0.98	6.38	10.77	0.85	14.39	28.21
